# Development of a Clonal and High-Yield Mammalian Cell Line for the Manufacturing of a Hyperactive Human DNase I with Extended Plasma Half-Life Using PASylation^®^ Technology

**DOI:** 10.3390/pharmaceutics16070967

**Published:** 2024-07-22

**Authors:** Serge M. Stamm, Roland Wagner, Dietmar A. Lang, Arne Skerra, Michaela Gebauer

**Affiliations:** 1 Rentschler Biopharma SE, Erwin-Rentschler-Str. 21, 88471 Laupheim, Germany; serge.stamm@rentschler-biopharma.com (S.M.S.); r.wagner@tu-braunschweig.de (R.W.);; 2 XL-Protein GmbH, Lise-Meitner-Str. 30, 85354 Freising, Germany; 3 Lehrstuhl für Biologische Chemie, Technische Universität München, Emil-Erlenmeyer-Forum 5, 85354 Freising, Germany

**Keywords:** Dornase alfa, half-life, NET, PASylation, Pulmozyme, stroke, thrombolysis

## Abstract

Cumulative evidence from several pre-clinical studies suggests that restoration of plasma DNase activity in a thrombo-inflammatory state may improve clinical outcomes. Following injury, hyperactivated immune cells release large amounts of granular proteins together with DNA, which often accumulate in the surrounding environment in so-called neutrophil extracellular traps (NETs). Degradation of excess NETs by systemic DNase administration offers a promising therapeutic approach to ameliorate inflammation and dissolve intravascular clots. In order to expand the therapeutic utility of human DNase I, a variant of the enzyme was developed that has both a prolonged systemic half-life and a higher catalytic activity compared to Dornase alfa (Pulmozyme^®^), the recombinant form of DNase I approved for inhaled therapy of cystic fibrosis. The hyperactive enzyme was “PASylated” by genetic fusion with a strongly hydrophilic and biodegradable PAS-polypeptide to increase its hydrodynamic volume and retard kidney filtration. A stable TurboCell™ CHO-K1-based cell line was generated which is suitable for the future production of PASylated DNase I according to good manufacturing practice (GMP). Furthermore, a robust bioprocess strategy was devised and an effective downstream process was developed. The final protein product is characterized by excellent purity, favorable physicochemical properties, a 14-fold higher DNA-degrading activity than Dornase alfa and a sustained pharmacokinetic profile, with a 22-fold slower clearance in rats.

## 1. Introduction

When applying endogenous proteins as therapeutic entities to correct an acquired or inherited deficiency of a native protein or to alter a disease process, these molecules are often far from ideal to be used as drugs [1]. Apart from limitations in physicochemical stability and/or immunogenicity, proteins are often rapidly cleared from the body and must be dosed frequently to maintain a therapeutic effect.

In the beginning of the last century, patients suffering from the genetic disorder cystic fibrosis (CF) rarely survived early childhood. However, the inhalation of nebulized recombinant human deoxyribonuclease (Dornase alfa, Pulmozyme^®^; Genentech, South San Francisco, CA, USA) increased life expectancy drastically to almost 40 years in 2010 [2]. Even though CF is a multi-systemic disease, live-threatening complications start in the respiratory system, where a chronic inflammation of airway epithelial tissue leads to the release of large amounts of double-stranded DNA. Consequently, breathing becomes difficult as this hyper-viscous mucus blocks the smaller airways. Dornase alfa, which was approved by the FDA in 1993 [3,4], is an almost identical version of the human endogenous enzyme, a monomeric 260-amino acid glycoprotein that catalyzes the hydrolysis of DNA phosphodiester bonds by a single-strand nicking mechanism in the presence of Ca^2+^ and Mg^2+^ [5]. The resulting decrease in molecular size of DNA reduces the viscosity of mucus [3]. In fact, daily administered Dornase alfa has a positive effect on mucus clearance and considerably improves lung function [6], thus justifying the original rationale for its development as a biopharmaceutical.

Mounting evidence over the past decade has furthermore supported the notion that recombinant DNase I could be useful for other therapeutic implications, such as infection or inflammation, where critical amounts of cell-free circulating nucleic acids are released. This often happens in concert with the liberation of neutrophil-derived proteins, together forming the structural backbone of so-called neutrophil extracellular traps (NETs; [7,8]). Initially described as an antimicrobial effector function of frustrated neutrophils to trigger inflammatory cascades in response to invaders [7], the filamentous web-like structure of NETs was recently identified as a potential therapeutic target in ongoing inflammations [9]. In addition, several studies have suggested that the lysis of thrombi with high DNA content is more successful when complementing the standard therapy with exogenous DNase I [10,11,12,13]. Notably, the level of extracellular DNA in blood was found to be positively associated with an inferior clinical outcome in a growing number of thrombo-inflammatory diseases such as sepsis [14,15], ischemic stroke [10], thrombosis [16,17,18], traumatic brain injury [19,20], myocardial infarction [21] and, most recently, SARS-CoV-2 infection [22,23].

While under physiological conditions the level of extracellular DNA in blood is low, due to its continuous digestion by endogenous DNases [24,25], elevated DNA levels under conditions of acute inflammation often correlate with diminished serum DNase I activity [20,23,26,27,28], probably caused and exacerbated by the post-injury release of DNase-specific as well as plasminogen-specific inhibitors [20,29,30]. Given that circulating endogenous DNA activates and perpetuates the immune response and, thereby, promotes organ and vascular damage, the timely depletion of extracellular DNA and NETs plays a role for tissue homeostasis and could also avoid the presentation of self-antigens [31,32].

However, in a clinical setting, the rather short plasma half-life of human DNase I of 3 to 4 h [33] likely limits the repurposing of Dornase alfa for the parenteral route of administration. By creating a PASylated human DNase I, we set out to develop a longer-acting version with increased half-life and also enhanced enzymatic activity to boost its pharmacological effect [34]. PASylation^®^ is a proprietary technology that makes use of a synthetic unstructured polypeptide chain which increases the apparent molecular size of its fusion partner [35], thereby retarding kidney clearance in a manner analogous to conjugation with polyethylene glycol (PEG). The PAS-biopolymer is solely composed of the three natural amino acids proline (P), alanine (A) and serine (S). In contrast to the chemical polymer PEG [36], which recently gained attention because of patient safety issues [37], PAS is less immunogenic and it is biodegradable [38]. Here, its favorable properties were combined with the clinically validated human enzyme DNase I using recombinant DNA technology by generating a constitutively expressing CHO cell line for a GMP-ready bioprocess including effective purification. Finally, the potency of the PASylated DNase I product was confirmed in a first preclinical study via administration to Wistar rats through the intravenous route.

## 2. Materials and Methods

### 2.1. Construction of the Vector for Recombinase-Mediated Cassette Exchange

A modified vector backbone compatible with our proprietary TurboCell™ line was used for cloning and integration of a single copy of the coding region for the PASylated hyperactive DNase I into the genome via recombinase-mediated cassette exchange (RMCE) [39]. For construction of the coding region, the 260-residue amino acid sequence of Dornase alfa (DrugBank Accession No. DB00003) was back-translated into a synthetic gene with codon optimization for CHO cells (Geneart, Regensburg, Germany), including the single amino acid substitution N74K [40]. At the N-terminus of DNase I, a pair of *Sap*I restriction sites in reverse orientation was incorporated to allow the subsequent in-frame cloning of a 400 residue PAS-gene cassette having compatible sticky ends (XL-protein, Freising, Germany). The final TurboCell™ RMCE plasmid harbored a PAS-DNase I expression cassette that includes a cytomegalovirus-immediate early promotor, a Kozak consensus sequence for optimal initiation of translation as well as a signal sequence for efficient secretion of the recombinant protein and is flanked upstream and downstream by unique recombinase recognition sites. The plasmid sequence was verified by analytical restriction digest and DNA sequencing prior to its use for transfection.

### 2.2. CHO Cell Line Development and Clone Selection

A TurboCell™ line based on CHO K1 [39,41] that bears a genomic acceptor cassette at a conserved spot within its genome (Rentschler Biopharma, Laupheim, Germany) was co-transfected with the RMCE vector from above, together with a plasmid coding for a recombinase. Upon transient expression of the recombinase, its pair of recognition sequences within the genome is recombined with the corresponding recognition sequences of the RMCE vector. This results in loss of the genomic acceptor cassette which otherwise effects constitutive expression of the green fluorescent protein (GFP) [39]. Resulting producer cells (GFP-negative) were separated from non-producer cells (GFP-positive) via fluorescent-activated cell sorting (FACS) using an SH800S instrument (Sony Biotechnology, San Jose, CA, USA). After sorting into 384-well plates, the individual cells were cultured at 36.5 °C under 5% *v*/*v* carbon dioxide atmosphere for 14 days. Afterwards, a selective expansion cascade from the 384-well stage to (i) 175 clones in two 96-well plates, then to (ii) the top 48 clones in 24-well plates and, finally, to (iii) the top 20 clones cultured in 50 mL conical centrifuge tubes (Corning, Corning, NY, USA) was accomplished using a three-to-four-day “passage by dilution” scheme as soon as sufficient cells for a volume increase were available. The criteria for selection involved a comparison of cell growth characteristics, including viability and viable cell density (VCD), utilizing a Guava easyCyte flow cytometer (Merck, Darmstadt, Germany). In addition, levels of secreted PASylated DNase I were determined by means of the PicoGreen activity assay. Chemically defined serum-free PowerCHO5 Medium (Lonza, Basel, Switzerland), supplemented with 6 mM L-glutamine (Thermo Fisher, Karlsruhe, Germany), was used for the adaptation of clones from the 24-well stage onwards. After a total of nine passages, 5.0 × 10^6^ viable cells were frozen in 1 mL conditioned medium supplemented with 10% *v*/*v* dimethyl sulfoxide (DMSO) and stored at −70 °C.

Expression and growth characteristics of single clones were further evaluated in parallel in small-scale fed-batch cultivations using shake flasks. Therefore, an inoculum of 0.6 × 10^6^ viable cells per mL was subjected to a 14-day fed-batch cultivation in a volume of 30 mL PowerCHO5 Medium, supplemented with 1 mM L-glutamine, in 125 mL shake flasks at 35 °C. A bolus feeding scheme for each shake flask, on cultivation days 3, 5, 6 and 7, with each receiving 3 mL (corresponding to 10% *v*/*v* of the initial volume) IS CHO-CD F12.7 Feed (FujiFilm, Santa Ana, CA, USA), was followed by glucose supplementation to a concentration of 6 g L^−1^ each on day 10 and on day 12. Volume loss due to evaporation was determined gravimetrically and compensated for by adding water for cell culture applications. Cell counts and viabilities during the fed-batch cultivation were measured using a Vi-CELL XR Cell Viability Analyzer (Beckman Coulter, Brea, CA, USA). The cell culture supernatants were recovered on day 14 by centrifugation at 4500× *g*, followed by 0.2 µm sterile filtration.

### 2.3. SDS-PAGE and Western Blotting

Cell culture supernatants and protein samples were separated by reducing SDS-PAGE using NuPAGE 4–12% Bis-Tris Protein Gels with NuPAGE MOPS Running Buffer (Thermo Fisher). Gels were stained with Coomassie G-250 SimplyBlue SafeStain (Thermo Fisher). For Western blot analysis of culture supernatants, 5 µL of a 5-fold dilution from day 14 of the small-scale 30 mL fed-batch culture was subjected to SDS-PAGE. Then, semi-dry electro-blotting onto a PVDF membrane was conducted using the Lightning Blot Mini Transfer system (PerkinElmer, Waltham, MA, USA) and a Novex Semi-Dry Blotter (Invitrogen, Carlsbad, CA, USA) at 12 V for 25 min. The membrane was blocked for 1.5 h in 1× TBST (20 mM Tris, 150 mM NaCl, 0.1% *v*/*v* Tween-20, pH 7.4) supplemented with 3% *w*/*v* bovine serum albumin (BSA). First, the membrane was incubated with a 1:2000 dilution of an anti-DNase I antibody (ab113241; Abcam, Cambridge, UK) in 1× TBST and incubated overnight at 4 °C under agitation. After the membrane was thoroughly washed with 1× TBST, a 1:5000 dilution of a secondary horseradish-peroxidase (HRP)-conjugated goat anti-rabbit antibody (111-035-003; Jackson ImmunoResearch, Cambridgeshire, UK) was applied for 1 h at room temperature. Subsequent washing with 1× TBST was followed by development of the blot with the chemiluminescent ECL Western Blotting Substrate (Thermo Fisher).

For the Western blot analysis of rat plasma samples, 15 µL of a 1:10 dilution of pooled plasma from three animals was subjected to SDS-PAGE under reducing conditions, followed by transfer of the separated proteins onto a nitrocellulose membrane using the iBlot Dry Blotting System (Invitrogen). The blotted membrane was blocked with 5% *w*/*v* non-fat dry milk in phosphate-buffered saline (PBS) supplemented with 0.5% *v*/*v* Tween 20 (PBST) at room temperature for 45 min under agitation, washed three times with PBST and then incubated with a 1:4000 dilution of Avi-PA(S)-Mab^®^ 1.1 [42] in PBST for 2 h. After three additional washing steps with PBST, the membrane was incubated for 1 h with a 1:4000 dilution of polyclonal goat anti-mouse IgG (H+L) alkaline phosphatase (AP) conjugate (Abcam). The membrane was washed three times, and signals were then visualized using the chromogenic alkaline phosphatase substrate BCIP/NBT (Thermo Fisher).

### 2.4. Protein Sequencing by Edman Degradation

To verify the cleavage of the signal sequence and integrity of the N-terminal PAS-sequence, a purified protein sample was first separated by SDS-PAGE and then transferred onto a PVDF membrane (Thermo Fisher) via electroblotting for 1.5 h at 15 V and 1000 mA on ice, using 50 mM Na-borate pH 9.0 supplemented with 10% *v*/*v* methanol as transfer buffer. Transferred proteins were detected by staining the membrane with 0.025% *w*/*v* Coomassie R-250 in 40% *v*/*v* methanol for 5 min at room temperature (RT) and subsequent de-staining in 50% *v*/*v* methanol. Bands for PASylated-DNase I were excised and analyzed by Edman degradation on an ABI Procise 494 instrument (Applied Biosystems, Waltham, MA, USA) operated by the Proteome Factory (Berlin, Germany).

### 2.5. Cell Cultivation in Bioreactors

For the production of PASylated DNase I at preparative scale and evaluation of the upstream cultivation platform we utilized a 2.7 L DASGIP Parallel Bioreactor System (Eppendorf, Hamburg, Germany). Continuous and cell-specific feeding of the TurboCell™ clone #137 were compared in duplicates during a 16-day fed-batch experiment at 35 °C. A viable cell density of 0.8–1.0 × 10^6^ per mL in 1000 mL PowerCHO5 medium, supplemented with 1 mM L-glutamine, was transferred for seeding to each of the four bioreactors, and the pH was controlled between pH 6.8 and 7.2 using a CO_2_ sparger and 1 M NaHCO_3_ solution. Cells were fed from day 2 to day 10 with either (i) continuous feeding of 2.1 mL/h or (ii) cell-specific feeding of 7.5 pg cell^−1^ d^−1^ of a chemically defined feed solution containing glucose, L-glutamine, antibiotics and antimycotics (FujiFilm). From day 7 till harvest on day 16 a daily glucose bolus feed was applied for both setups to restore glucose concentration to 6 g L^−1^. Glucose concentrations were measured using a Cedex Bio HT Analyzer (Roche, Mannheim, Germany). Cells were counted and viabilities were measured using a Vi-CELL XR Cell Viability Analyzer (Beckman Coulter, Brea, CA, USA). Finally, cells were harvested on day 16 by centrifugation at 4500× *g*. The cell-free supernatant was sterilized by 0.2 µm filtration and stored at −20 °C.

### 2.6. DNA-PicoGreen Assay

Recombinant human DNase I activities in cell culture supernatants or rat plasma samples were measured using a hydrolytic DNase activity assay based on the fluorescent DNA-intercalating dye PicoGreen [43]. A 20 µg/mL substrate stock solution of salmon testis dsDNA (Sigma, St. Louis, MO, USA) was prepared in CM buffer (25 mM HEPES/NaOH pH 7.5, 4 mM CaCl_2_, 4 mM MgCl_2_, 0.01% *w*/*v* thimerosal). Dornase alfa (Genentech) was used as a standard, and all samples were diluted in buffer C (0.1% *w*/*v* BSA, 0.05% *v*/*v* Tween 20 in buffer CM) to cover the linear concentration range of the assay, between 10 and 1000 ng mL^−1^ of DNase I. Buffer C was used as blank. Measurements were performed in 96-well black fluorescence microtiter plates (Corning) containing a mixture of 10 µL (0.2 µg) substrate DNA solution, 10 µL diluted protein sample and 80 µL buffer CM per well. Plates were sealed and incubated for 30 min at 37 °C, then 100 µL of a 1:200 dilution of PicoGreen dsDNA quantitation reagent (Invitrogen) in TE buffer (10 mM Tris/HCl, 1 mM EDTA, pH 7.4) was added to generate the fluorescence signal and stop the enzymatic reaction. The plate was incubated for 5 min at room temperature in the dark. Fluorescence signals were quantified in a SpectraMax M5e microtiter plate reader (Molecular Devices, San José, CA, USA) at 480 nm excitation and 520 nm emission wavelengths. Unknown sample concentrations were calculated by comparison to the standard curve. Of note, while Dornase alfa was used for the analysis of cell culture supernatants, due to the higher activity and molecular weight of PASylated DNase I the purified PASylated DNase I was used for the analysis of rat plasma samples.

The DNA-PicoGreen assay was also used to compare equimolar concentrations of purified recombinant human DNases in the presence of 25 mM HEPES/NaOH pH 7.3, 1 mM MgCl_2_, 2.5 mM CaCl_2_ and 140 mM NaCl, which matches the ionic strength and pH of biological solutions [44]. Therefore, 2.5 µg mL^−1^ of salmon testis dsDNA (Sigma) was mixed with serial dilutions of either Dornase alfa or PASylated hyperactive DNase I and incubated for 30 min at 37 °C. A 50 µL aliquot of each reaction was transferred to a 96-well black fluorescence microtiter plate, and enzymatic activity was immediately stopped by the addition of 150 µL per well of a freshly prepared 1:300 dilution of Pico488 (Lumiprobe, Hannover, Germany) in 2.5 mM EDTA/NaOH pH 8. Samples were allowed to equilibrate at room temperature for 5 min in the dark before fluorescence signals were quantified as described above. Unspecific background binding was minimized by using low DNA/protein binding plasticware throughout the assay.

### 2.7. Protein Purification

All purification steps were performed on an ÄKTA explorer system (Cytiva, Freiburg, Germany). Initially, the conductivity of the cell-free culture supernatant was adjusted to ≤8 mS cm^−1^ by adding an equivalent volume of CB chromatography buffer (20 mM MES, 20 mM HEPES, 20 mM Na-acetate, 20 mM NaCl, 1.0 mM CaCl_2_, 1.5 mM MgCl_2_, adjusted with NaOH to pH 7.5). 75 mL of the adjusted supernatant was loaded on a Nuvia HP-Q anion exchange column (5 mL bed volume; Bio-Rad, Feldkirchen, Germany) that had been pre-equilibrated with CB, and the column was washed until the baseline was reached, followed by applying a linear concentration gradient from 0 to 1 M NaCl. Fractions containing the PASylated hyperactive DNase I were identified by SDS-PAGE and pooled. Then, the protein preparation was adjusted to a conductivity of 148–152 mS cm^−1^ with a 4 M (saturated) stock solution of ammonium sulfate. The sample was sterile-filtered (0.45 µm and 0.2 µm Millex Syringe Filters; Merck) and loaded onto a pre-equilibrated 5 mL TOYOPEARL Phenyl-650M column (Tosoh Bioscience, Sursee, Switzerland) for hydrophobic interaction chromatography (HIC). The column was washed with equilibration buffer (CB supplemented with 1.2 M ammonium sulfate, pH 7.5) until the baseline was reached, and the PASylated DNase I was eluted using a descending ammonium sulfate concentration gradient approaching 100% CB. Fractions containing the PASylated DNase I were identified by SDS-PAGE, pooled, concentrated by an ultrafiltration device (Pierce Protein Concentrators PES, 50K MWCO; Thermo Fisher) and desalted by dialysis against CB (Slide-A-Lyzer 10K MWCO Dialysis cassettes; Thermo Fisher). For polishing, the protein sample was subjected, in flow-through mode, to a multimodal cation exchange chromatography matrix, Capto MMC (Cytiva), which had been pre-equilibrated to pH 7.5 with buffer CB. The recovered flow-through from this step was adjusted to pH 8.0 by the addition of CB adjustment buffer (20 mM Na-acetate, 20 mM MES, 20 mM HEPES, 20 mM NaCl, 1 mM CaCl_2_, 1.5 mM MgCl_2_, 500 mM Tris, adjusted with NaOH to pH 9.2) and loaded onto multimodal Capto Core 400 resin (Cytiva), pre-equilibrated with CB adjusted to pH 8.0. At pH 8.0, residual (mainly acidic) host cell proteins ≤ 400 kDa were bound to the positively charged resin, whereas the PASylated DNase I (calculated pI 4.6) was recovered in the flow-through. The purified protein was finally dialyzed against the formulation buffer of Dornase alfa (150 mM NaCl, 1 mM CaCl_2_, nominal pH 6.3) [45] for further characterization. Protein concentrations were determined by absorption measurement at 280 nm using a calculated molar extinction coefficient of 46,090 M^−1^ cm^−1^ [46]. Endotoxin was quantified using a kinetic turbidimetric test with Limulus amebocyte lysate (LAL) according to the European Pharmacopoeia (Ph. Eur.), chapter 2.6.14.

### 2.8. Size Exclusion Chromatography

Purified proteins were analyzed by size exclusion chromatography (SEC) on a 24 mL Superdex 200 Increase 10/300 GL column (Cytiva) equilibrated with PBS (115 mM NaCl, 16 mM Na_2_HPO_4_, 4 mM KH_2_PO_4_) at RT. A sample volume of 200 µL for both Dornase alfa (1 mg mL^−1^) and purified PASylated DNase I (2 mg mL^−1^) was applied at a flow rate of 0.5 mL min^−1^. Elution volumes and apparent protein sizes were estimated by interpolation from a half-logarithmic calibration line utilizing a set of protein standards (Merck) with known molecular masses in PBS. The column void volume was determined using Blue Dextran.

### 2.9. Dynamic Light Scattering

Dynamic light scattering (DLS) measurements were performed on a Zetasizer nano S instrument (Malvern, Kassel, Germany) using a 3 mm quartz cuvette (Hellma, Mühlheim, Germany) and the internal standard operating procedure “Protein Size at 25 °C”. Proteins were first subjected to SEC in PBS before the eluate was concentrated by ultrafiltration (Vivaspin 500; Sartorius, Göttingen, Germany) to 1.1 mg mL^−1^ (29 µM) for Dornase alfa and to 1.7 mg mL^−1^ (27 µM) for PASylated DNase I. Data represent the Z-average mean hydrodynamic diameter [nm] of at least 3 independent runs, all meeting the internal quality criteria. The software internal function “Molecular Weight Estimate” for globular proteins was used to convert the hydrodynamic diameter to molecular weight. For thermal protein unfolding experiments, proteins were dialyzed against formulation buffer (150 mM NaCl, 1 mM CaCl_2_, nominal pH 6.3), concentrated to 1 mg mL^−1^ by ultrafiltration and subjected to an automated 2 °C incremental temperature ramp from 20 °C to 90 °C, with a 180 s equilibration period at each step, using the “Trend Temperature Size” function of the device and with “water/PBS” set as buffer system. The temperature at the midpoint of the unfolding transition, T_m_, was calculated from the thermal denaturation experiments by fitting the data using non-linear least-squares regression (KaleidaGraph version 5.0.4; Synergy Software, Reading, PA, USA) and an equation for a two-state model of unfolding as previously described [47].

### 2.10. Circular Dichroism Spectroscopy

Circular dichroism (CD) spectroscopy measurements were performed as previously published [48]. In brief, proteins were dialyzed against 50 mM K_2_SO_4_, 20 mM KP_i_ pH 7.5 and diluted to a final concentration of 25 µM for Dornase alfa and of 10 µM for PASylated DNase I. Spectra were recorded at 20 °C from 190 nm to 250 nm using a J-810 spectropolarimeter (Jasco, Easton, MD, USA) with a 0.1 mm path length quartz cuvette (Hellma). After correction for the buffer blank, normalized spectra were smoothed using the instrument software (Spectra Viewer with J-800 control drive version 1.24.00). The molar ellipticity was calculated according to the equation Θ_M_ = Θ_obs_/(c × d), with Θ_obs_ representing the measured ellipticity, c the concentration of the protein [mol L^−1^] and d the cuvette path length [cm].

### 2.11. Pharmacokinetic Study in Rats

Animal experiments were conducted at the Aurigon Toxicological Research Center (Dunakeszi, Hungary) following the 2010/63/EU guideline for animal welfare. Nine female Han Wistar rats (8–9 weeks old) were divided into three groups (N = 3 × 3) and housed in a pathogen-free environment under controlled conditions of 22 ± 3 °C, 50 ± 20% relative humidity and a 12/12 h light/dark cycle. Animals were injected intravenously (i.v.) with 5.33 mg per kg body weight of purified, endotoxin-free (≤0.11 endotoxin units per injection) PASylated hyperactive DNase I. Blood samples (500 µL) were collected in Li-heparin tubes at various time points: 0.5 h, 4 h and 24 h for group I; 1 h, 8 h and 48 h for group II; 2 h, 12 h and 96 h for group III. Plasma was prepared by centrifugation at 3000× *g* for 10 min at RT, and the supernatant was stored at −20 °C. Plasma concentrations of PASylated DNase I were quantified by the DNA-PicoGreen activity assay as described above. Endogenous DNase activity from an untreated rat was evaluated as blank value. PK analysis was performed using Phoenix WinNonlin software version 6.1 (Pharsight, St. Louis, MO, USA). According to the Akaike Information Criterium (AIC) of the software as well as graphical assessment of the model assumptions, the plasma concentration/time data were best described by a two-compartment model with bolus input, first-order output and micro-constants as primary parameters: C(t) = Ae^−αt^ + Be^−βt^, where *A* and *B* are concentration unit-coefficients and *α* and *β* are exponential coefficients. Data were weighted with the reciprocal of the squared predicted concentration (1/$Y_{obs}^{2}$).

## 3. Results

### 3.1. Protein Design

For a broader therapeutic applicability of human DNase I, we aimed at (i) prolonged half-life in vivo, which was achieved by fusion with an expanded amino acid-/based biopolymer (PASylation) and (ii) enhanced catalytical activity under physiological conditions. Engineered variants of DNase I that are catalytically effective at substantially lower concentrations are known from the literature [49]. However, the constitutive overexpression of these hyperactive, salt- and actin-resistant mutants in cell lines during biopharmaceutical manufacturing is challenging, as they appear to be toxic for the host cell [50].

Naturally, within the body, the hydrolytic activity of endogenous DNase I is tightly regulated by binding to actin [51,52], a principal component of the cytoskeleton, as any mislocalization of this enzyme to the nucleus, e.g., by entering the cell via diffusion from outside the cell or by release from intracellular stores, would lead to premature cell death [50,52]. Therefore, we introduced the single amino acid substitution Asn74Lys, which is known to render DNase I hyperactive while still being susceptible to cellular actin inhibition [40]. This mutant of DNase I (N74K) was genetically fused to a PAS polypeptide comprising 400 amino acid residues, which had previously been shown to increase the hydrodynamic volume of proteins to approximately the same extent as conjugation with a 20–30 kDa high molecular weight PEG [35]. Installed at the N-terminus, the PAS polymer is located at a site which is not involved in DNA binding or hydrolysis [5].

### 3.2. Cell Line Development

For the recombinant production of the PASylated high-activity enzyme, we made use of our engineered Chinese Hamster Ovary K1 cell-based platform technology (TurboCell™). This system allows for the generation of highly stable recombinant cell lines by site-directed integration into a specific expression hotspot of the genome with the help of a recombinase-mediated cassette exchange (RMCE) [39]. To this end, the synthesized nucleotide sequence, encoding the hyperactive human DNase I genetically fused at its N-terminus with the PAS sequence as well as a signal peptide, was cloned on a transfer vector providing a cytomegalovirus (CMV) promoter and two different recombinase recognition sequences flanking the transgene cassette [39]. The resulting vector and a helper plasmid encoding the recombinase system [39] were then co-transfected into the TurboCell™ host cell line. Successful site-directed integration of the transgene by cassette exchange was indicated by the loss of the gene for the green fluorescent protein (GFP) that served as negative selectable marker, pre-localized on the chromosome, which allowed for the efficient separation from non-recombined cells by fluorescence-activated cell sorting (FACS).

14 days after FACS sorting, a total of 175 single-cell derived clones were recovered at the 384-well stage. In order to identify the best producers, the number of candidates was further narrowed, in two steps, to a set of 20 clones with optimal growth, viability and expression characteristics, as judged from flow cytometry and a PicoGreen-based DNase activity assay, which were expanded to 30 mL fed-batch cultivation. While all these clones demonstrated high viability over the 14-day period of fed-batch cultivation, with viable cell densities (VCD) of up to 22 × 10^6^ mL^−1^, eight clones exhibited increased specific protein productivity in the range of 4.6–9.3 pg d^−1^ cell^−1^ as quantified by the PicoGreen assay with samples of the culture supernatant (Table 1).

**Figure 1:**
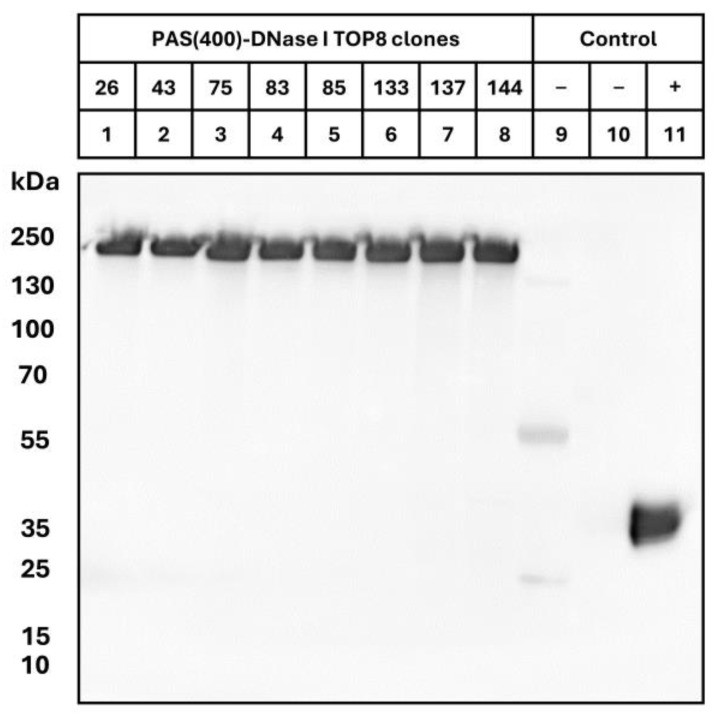
Western blot analysis of crude cell supernatants from stably transfected CHO TurboCell™ clones secreting recombinant PASylated hyperactive DNase I. Fed-batch cell culture supernatants after 14 days of cultivation (30 mL) were separated by reducing SDS-PAGE and blotted onto a PVDF membrane. DNase I was detected by a rabbit anti-human DNase I antibody, followed by an HRP-conjugated goat anti-rabbit antibody. Lanes 1–8: 1 µL of each culture supernatant; lane 9: 1 µL trastuzumab culture supernatant (negative control); lane 10: 1 µL untransfected TurboCell™ (mock) culture supernatant; lane 11: 25 ng Dornase alfa. Note that PASylated proteins in general migrate in SDS-PAGE at an apparently higher molecular weight than expected from their true mass [31]. N-linked carbohydrate heterogeneity at Asn18 and Asn106 in Dornase alfa causes the protein to migrate as multiple bands with an average MW of 37 kDa [3] while the molecular weight predicted from the amino acid sequence is 29.3 kDa. Low background signals observed for the control (lane 9) can be attributed to cross reactivity of the secondary antibody with heavy and light chains of trastuzumab. Original images of the figure can be found in Supplementary Materials.

The integrity of the cloned coding region and gene product for the top eight producer cell lines after 14 days of cultivation were verified by an anti-DNase I Western blot, revealing the presence of a uniform product of the PASylated enzyme in all cell culture supernatants (Figure 1). Clonal cell line #137, which showed the highest final recombinant protein titer (438 mg L^−1^) as well as cell-specific productivity (9.3 pg d^−1^ cell^−1^) under the growth conditions tested, was chosen as the preferred candidate for process development in a bench-top bioreactor. In addition, the identity of the first eight N-terminal amino acids of the final product, i.e., mature PASylated DNase I, was confirmed by Edman protein sequencing (SPAAPAPA).

### 3.3. Controlled Bioreactor Setup for Clone #137

**Figure 2:**
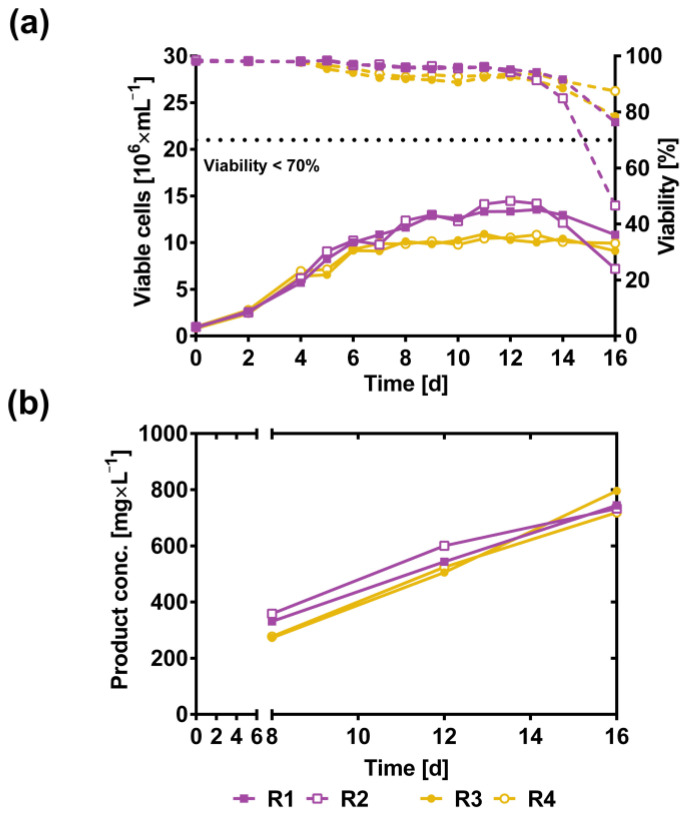
Data from fed-batch cultures in a 2.7 L controlled bioreactor with the TurboCell™ CHO clonal cell line #137 expressing PASylated hyperactive DNase I. (**a**) Viable cell density (solid lines) and viability (dashed lines) over a 16-day fed-batch cultivation. Cultivation was performed in duplicate with either continuous feeding (bioreactors R1 and R2) or cell-density dependent feeding (bioreactors R3 and R4). 70% viability was applied as harvest criterion (horizontal dotted line), except for R2. (**b**) Product concentration titers were measured off-line using the PicoGreen DNase I activity assay on cultivation days 8, 12 and 16. The protein titer (mass of the PAS polypeptide included) was calculated by using a standard curve with known concentrations.

The clonal cell line #137 demonstrated the highest cell-specific productivity in shake flask culture and, thus, was employed to establish a scalable fed-batch process in a controlled bioreactor environment. Two different strategies for substrate supply, (i) continuous and (ii) cell-specific feeding, were tested in parallel in 2.7-L benchtop bioreactors to assess the effect on cell growth and product titer. VCDs of up to 15 × 10^6^ mL^−1^ for continuous feeding (bioreactors R1 and R2) and up to 10 × 10^6^ mL^−1^ for cell-specific feeding (bioreactors R3 and R4) were observed (Figure 2a), which was in good correlation with our experience from other biologics produced using the TurboCell™ technology. Cell viabilities remained >80% for both feeding schemes until day 14 but dropped significantly on day 16, at the time of harvest. While for three of the four bioreactors the harvest criterion (<70% viability) was met at this time point, the VCD in bioreactor 2 (continuous feeding) had declined already to 47%. Possibly, the higher initial cell density observed in this bioreactor (Figure 2a) may have resulted in an elevated accumulation of toxic cell metabolites. Even though lower VCDs were generally observed for the cell-specific feeding scheme, comparable product titers were achieved for both strategies (Figure 2b), ranging from 733 (R2) to 796 mg L^−1^ (R3).

### 3.4. Recombinant Protein Purification

**Figure 3:**
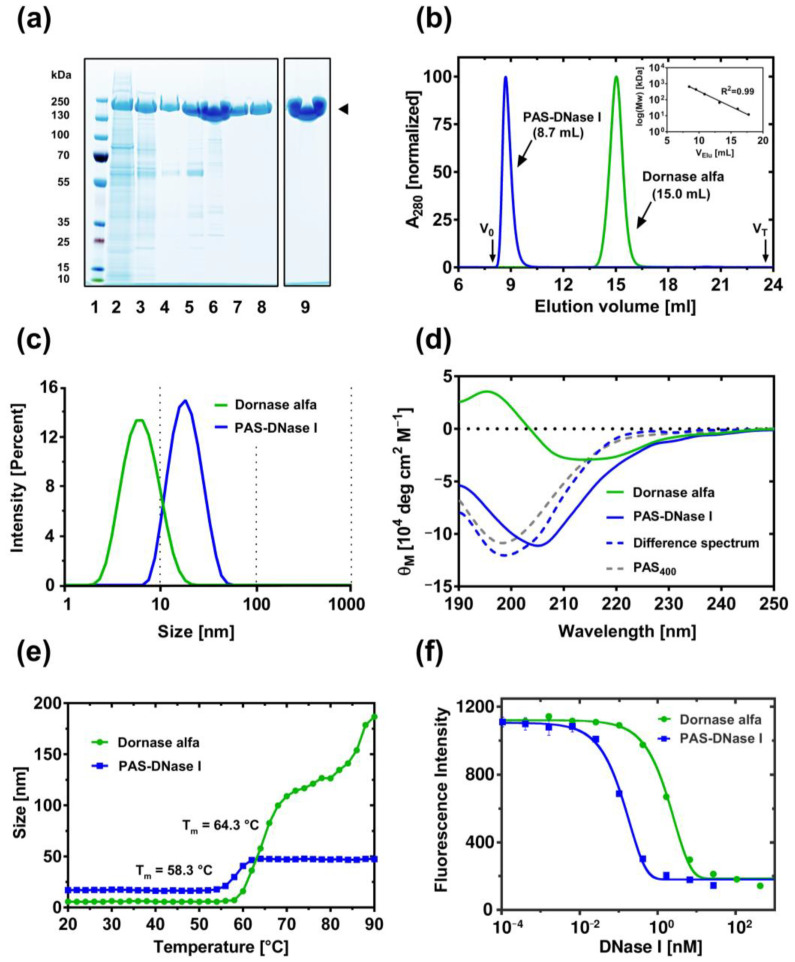
Biochemical and biophysical characterization of PASylated hyperactive DNase I. (**a**) SDS-PAGE analysis of the downstream purification process. Coomassie-stained reducing SDS-PAGE (4–12%). Lane 1: MW standard; lane 2: harvested cell culture supernatant from the bioreactor of clone #137; lane 3: AEX elution: lane 4: HIC elution; lane 5: CEX load after concentration and dialysis; lane 6: CEX flow-through (concentrated); lane 7: early flow-through from multimodal chromatography; lane 8: late flow-through; lane 9: concentrated protein solution of the final protein preparation (63 µg). Note: All lanes result from a single gel image, lane 9 has been re-arranged when preparing the figure. (**b**) Analytical SEC. Elution profiles (overlay) of PASylated hyperactive DNase I and Dornase alfa on Superdex Increase 200 10/300 GL. Both proteins eluted as a single symmetrical peak while PASylated DNase I showed a significantly lower elution volume (8.7 mL) compared to Dornase alfa (15 mL). V_0_, exclusion volume (Blue Dextran); V_T_, column volume. Inset: Half-logarithmic calibration curve with a set of standard proteins (cytochrome C, bovine serum albumin, β-lactamase, β-amylase, apoferritin and thyroglobulin). (**c**) Measurement of the hydrodynamic diameter for Dornase alfa and the PASylated hyperactive DNase I by DLS. Results are shown as average of three individual measurements in Dornase alfa formulation buffer. The intensity-weighted mean hydrodynamic size is reported in Table 2. (**d**) Far-UV CD spectra of Dornase alfa and PASylated hyperactive DNase I. The molar difference spectrum of both proteins reveals a characteristic minimum at 200 nm similar to the spectrum of the isolated PAS_400_ polypeptide published before [35]. (**e**) Thermal unfolding and aggregation of Dornase alfa and PASylated hyperactive DNase I monitored by DLS. Comparison of the Z-average diameter in steps of 2 °C. Note: The larger size of the PASylated hyperactive DNase I compared with the reference protein, Dornase alfa, in the temperature range below T_m_ is simply an effect of the attached PAS-biopolymer with its voluminous random coil structure. (**f**) Fluorescence-based DNA hydrolytic activity assay of PASylated hyperactive DNase I and Dornase alfa under near-physiological buffer conditions. DNase I activities were quantified at 37 °C using the specified enzyme concentrations and dsDNA as substrate in HEPES/NaOH pH 7.3, 140 mM NaCl. The decrease in fluorescence of the intercalating dye Pico488 as a result of DNA degradation was monitored spectrophotometrically. Original images of (**a**) can be found in Supplementary Materials.

To assess the product quality of the secreted PASylated hyperactive DNase I, the recombinant protein was purified from the clarified cell-culture supernatant by, first, anion exchange chromatography (AEX) and, then, hydrophobic interaction chromatography (HIC), which significantly reduced the proportion of host cell proteins (Figure 3a). The resulting protein preparation was further polished by applying a cation exchange chromatography (CEX) in flow-through mode and, finally, a multimodal chromatography on Capto Core 400 (Figure 3a, Lane 6–8), which is a dual-function matrix for the purification of large biomolecules, having a 400 kDa molecular weight cut-off [53]. Owing to the large increase in the hydrodynamic molecular volume of DNase I caused by the PAS-tag, the purified enzyme was collected from the Capto Core flow-through, while residual smaller host cell impurities remained effectively bound within the anionic ligand core of the matrix (Figure 3a, lanes 7 and 8). The PASylated DNase I purified by this sequence ultimately resulted in a homogenous monomeric protein preparation with >95% purity, as documented by SDS-PAGE (Figure 3a, lane 9) and analytical SEC (Figure 3b), which proved to be suitable for further biophysical characterization and, eventually, in vivo studies.

### 3.5. Biophysical and Enzymatic Characterization

The apparent molecular weight of the PASylated hyperactive DNase I was investigated by analytical SEC using a calibrated column, resulting in a 15-fold increase in hydrodynamic molecular volume compared to Dornase alfa (Figure 3b); thus, the N-terminal fusion with 400 PAS residues boosted the apparent molecular weight of the enzyme from 38 kDa to 581 kDa. This dramatic increase in size was further confirmed by dynamic light scattering (DLS), showing a 13-fold increase in size from 39 kDa to 494 kDa (Figure 3c), which corresponds to a 3-fold enlarged diameter of 17 nm vs. 5.7 nm (Table 2).

From the crystal structure it is known that human DNase I has a compact α/β fold [5]. Investigation of the secondary structure of Dornase alfa by circular dichroism (CD) spectroscopy (Figure 3d) revealed a positive band at 195 nm and, in particular, a broad minimum in the region between 208 nm and 220 nm, which is indicative of α-helical conformation. In contrast, the spectrum of PASylated DNase I revealed a strong negative band at 205 nm, followed by a shoulder, a characteristic feature of a protein CD spectrum dominated by random coil. By calculating the difference spectra of both proteins, the CD spectrum of the pure random coil conformation typical for the PAS polypeptide emerged as it was previously described [35].

While the PAS-biopolymer itself constitutes a biochemically inert amino acid sequence with high hydrophilicity [38], this biopolymer in fusion with proteins has been repeatedly shown to positively impact the stability and solubility of its fusion partner [54,55]. Thermal unfolding, which is accompanied by a large increase in hydrodynamic volume of the polypeptide chain, represents an important parameter to evaluate the conformational stability of proteins. Hence, we applied DLS to monitor the change in size distribution of native DNase I (Dornase alfa) vs. the PASylated hyperactive DNase I when subjected to an ascending temperature gradient from 20 to 90 °C in the protein formulation buffer of Dornase alfa (Figure 3e). Compared to Dornase alfa, the PASylated hyperactive enzyme had a steeper transition from its native conformation to the unfolded state while the melting temperature at the midpoint of the unfolding transition was just slightly reduced from 64.3 °C to 58.3 °C. Interestingly, only the unfolding of Dornase alfa revealed a second transition at higher temperature (above 80 °C). In fact, it appeared that native DNase I starts to form higher-order aggregates with increasing temperature, as evident from the steadily rising diameter values at the onset of protein unfolding/aggregation, whereas the PASylated protein resists further temperature-mediated protein aggregation, as seen in other cases before.

Substitution of the deamidation-prone residue Asn74 by the basic Lys side chain in the PASylated DNase I resulted in nuclease hyperactivity as shown by a head-to-head in vitro comparison of DNA digestion (Figure 3f). The rate of hydrolysis of high molecular weight double-stranded DNA into smaller fragments in a buffer system mimicking the physiological ionic strength [44] was 14-fold higher for the PASylated DNase I than for Dornase alfa as quantified by the decreasing fluorescence of a dsDNA intercalating dye.

### 3.6. Pharmacokinetic Analysis

**Figure 4:**
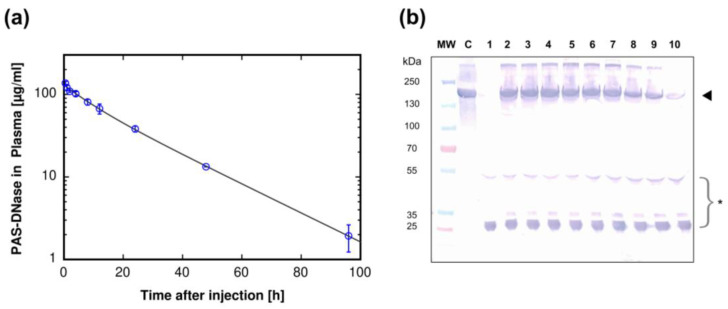
Pharmacokinetics of PASylated hyperactive DNase I in the rat. (**a**) Semi-logarithmic plasma concentration vs. time profile of the PASylated DNase I up to 96 h post i.v. injection in rats at a dose of 5.33 mg kg^−1^ body weight. Plasma concentrations of the fusion protein were quantified by the DNase activity-based PicoGreen assay. The bi-exponential decay of the protein concentration was fitted by a two-compartment model with first order elimination rate and the kinetics is described by C(t) = A*e*^−αt^ + B*e*^−βt^, with A = 37.2 µg mL^−1^, B = 92.9 µg mL^−1^, α = 0.117 h^−1^, β = 0.04 h^−1^. PK parameters were determined using WinNonlin software (see Section 2) and are listed in Table 3. Each point represents the mean ± standard deviation (N = 3). (**b**) Western blot analysis of rat plasma samples obtained at various sampling times after i.v. injection of PASylated hyperactive DNase I in (**a**). Equal volumes of rat plasma were subjected to reducing SDS-PAGE. PASylated hyperactive DNase I was detected by a murine anti-PAS Mab followed by a goat anti-mouse IgG (H+L) AP-conjugate. Lane MW: pre-stained molecular size standard; lane C: purified PASylated hyperactive DNase I (control); lane 1: plasma sample of untreated rat (blank); lanes 2–10: rat plasma samples at 0.5 h, 1 h, 2 h, 4 h, 8 h, 12 h, 24 h, 48 h and 96 h, respectively. Each time point represents a mixture of plasma collected from three animals. Arrowhead: PASylated hyperactive DNase I. * Some background staining of rat plasma immunoglobulins due to cross-reactivity of the polyclonal anti-mouse secondary antibody. Original images of (**b**) can be found in Supplementary Materials.

When administered intravenously, Dornase alfa is rapidly cleared from the blood, revealing a terminal plasma half-life of just around 3 h in rats [56]. Accordingly, we measured the effect of PASylation of the human hyperactive DNase I on the pharmacokinetic (PK) profile in rats (Figure 4a). For the in vivo PK assessment, nine Wistar rats received a single intravenous (i.v.) bolus dose of 5.33 mg purified fusion protein per kg body weight (b.w.), corresponding to a dose of 2.5 mg kg^−1^ b.w. of unmodified Dornase alfa, and this dose was well tolerated. Rat serum samples were collected at fixed time points, diluted in DNase formulation buffer and analyzed for enzymatic activity using the PicoGreen assay. We found a high analytical sensitivity of this assay, being able to detect the recombinant protein in plasma up to the last sampling point, at 96 h after injection, for which a mean plasma concentration of 1.9 µg mL^−1^ PASylated DNase I was determined. Endogenous DNase plasma concentrations from untreated rats appeared to be marginal (0.03 µg mL^−1^) and were neglected.

The resulting time course revealed a bi-exponential decline of PASylated DNase I plasma concentration over time and was best described by a two-compartment model after single i.v. bolus dose (Figure 4a). Assuming first-order elimination, the total clearance of PASylated hyperactive DNase I from the plasma was 2.04 mL h^−1^ kg^−1^ (Table 3) and, thus, more than 22 times lower than the reported clearance of 44.1 mL h^−1^ kg^−1^ for the unmodified recombinant DNase I (following i.v. administration of 1 mg mL^−1^ per kg b.w.) [56]. Notably, the steady state volume of distribution V_ss_ was smaller for the PASylated protein (46.4 mL kg^−1^ vs. 173 mL kg^−1^; [56]), which led to higher concentrations available in blood compared to the unmodified enzyme. Due to the much slower clearance of PASylated DNase I, its terminal plasma half-life in rats was considerably prolonged to 17.2 h (Table 3).

The plasma samples were also analyzed for the integrity of the PASylated fusion protein by Western blotting (Figure 4b). To this end, we used a monoclonal antibody that specifically detects an epitope within the PAS sequence [42]. A prominent protein band corresponding to the intact PASylated hyperactive DNase I was evident in plasma samples of treated rats, which was not detectable in naïve rats (Figure 4b). The intensity of this protein band remained almost unaltered up to 12 h after injection, before it started to gradually fade over time, which was in accordance with the gradual decrease of DNase I concentrations in plasma as measured by its activity in the PicoGreen assay described above.

## 4. Discussion

Various studies have demonstrated that the production of PASylated proteins in both prokaryotic and eukaryotic hosts generates novel proteins with expanded hydrodynamic volume and, therefore, improved pharmacokinetic properties [48,55,57,58,59]. Since DNase I requires specific N-glycosylation to be functional, a mammalian cell culture system such as the CHO TurboCell™ [39] offers the preferred manufacturing host. Nevertheless, successful overproduction of DNase I still remains challenging, as it bears the risk of toxic effects on the host cell, especially when its coding region is stably integrated into the genome [50]. As we also attempted to generate an enzyme with higher potency, we aimed for a careful pre-selection of mutations to gain hyperactivity without introducing stronger toxic effects on the mammalian expression host cell.

Indeed, a variant of PASylated DNase I which was equipped with the previously published N74K mutation for hyperactivity [40], together with a structurally disordered chain of 400 PAS residues [35], proved to be well suited for cell line development in terms of stable cell integration, viability, growth and secretory production yield. Based on the TurboCell™, the performance of the high-producer clone #137 was stable, thus providing a promising starting point for process development. The GMP-ready CHO TurboCell™ upstream platform bioprocess developed here—after comparing continuous with cell-specific feeding—led to high titers of PASylated hyperactive DNase I in a 2.7 L bioreactor setting with a cell-specific feeding scheme that also allows process scale-up. In fact, the product concentrations of up to 800 mg per L obtained for the PASylated hyperactive DNase I in our CHO host cell line were comparable to those previously described for the native recombinant human DNase I [50,60].

Notably, deamidation of Asn 74, which is located at the peripheral DNA binding interface of the enzyme, is a post-translational modification that has been reported to occur spontaneously during storage of Dornase alfa [45]. Although the structural integrity of DNase I does not seem to be compromised [61], with more than 65% of the formulated protein found deamidated, its stability and function are affected [45,62]. While the deamidation is accompanied by a change from neutral to negative charge on the surface area of DNase I, introduction of a positive charge at this position by mutation to Lys is assumed to stabilize the otherwise weak complex between DNase I and its negatively charged DNA substrate [49]. When comparing the purified PASylated DNase I mutant, obtained via the fed-batch production from clone #137, we observed a DNA-hydrolytic activity 14 times more potent than for Dornase alfa in physiological buffer. Thus, as expected from previous data on the activity of a series of engineered variants of DNase I [63], the enzyme becomes hyperactive by increasing the local electrostatic attraction towards DNA. Remarkably, the addition of the voluminous but conformationally flexible PAS biopolymer did not seem to hamper the enzymatic activity towards its macromolecular DNA substrate.

The expansion of the hydrodynamic volume of the enzyme due to its N-terminal PASylation was verified by SEC and DLS and, with both methods, a drastic increase compared to the unmodified DNase I was found. In fact, a 15-fold higher apparent molecular weight than determined for the wild-type enzyme was observed, which is largely attributed to the random coil conformation of the PAS polypeptide, whose unlimited number of potential conformations continuously fluctuate in the three-dimensional space—due to Brownian motion—around its fusion partner, thereby artificially increasing the apparent protein size [35]. Similar effects of size expansion due to PASylation were found in previous studies with other pharmacologically relevant proteins [35,48]. Unlike chemical PEGylation [36], the sharp protein elution peaks seen in SEC are common to PASylated biologics because there is no inherent size distribution of the genetically encoded polymer (in contrast to the chemical polymer PEG), which could lead to varying physicochemical properties, thus facilitating the purification process and product analytics.

Conformational and thermal stability are among the major factors affecting developability of a new biological entity, and native DNase I has been shown to be highly robust [61,64]. In general, introducing changes by protein engineering to modulate protein function may destabilize the enzyme, but the analytical techniques used in the present study indicate that, compared to Dornase alfa, the thermal stability of the enzyme is well preserved by introduction of the N74K mutation as well as fusion with the PAS polypeptide. Moreover, the PASylated enzyme seems to withstand the formation of higher molecular weight aggregates upon heating, which is indicative of stronger protein–protein repulsive interactions and therefore a higher colloid stability [65], a property that should contribute to minimizing any immunogenic potential [66].

The half-life of proteins in vivo is mainly determined by renal clearance, which depends on their apparent molecular size [67]. Globular proteins larger than 6 nm in diameter are typically retained by the glomerular filtration barrier of the kidney [68]. Thus, native DNase I with a measured diameter of 5.7 nm, i.e., below this threshold, is expected to show faster clearance from plasma than its PASylated version which is three times larger (17 nm). A terminal half-life of 3 h in rats was reported for the recombinant native DNase I following a single intravenous injection [56]. Interestingly, several PK parameters of DNase I were found to be dose-dependent in rats, such as the clearance (C_L_), steady state volume (V_ss_) and half-life of distribution ($t_{1/2}^{\alpha }$), whereas the terminal half-life, $t_{1/2}^{\beta }$, was unaffected [56]. Apparently, this behavior can be attributed to the non-covalent association of DNase I with specific binding proteins in plasma, one of them being identified as circulating actin. Apart from modulating the DNase activity, this also leads to a lower volume of distribution, as the actin complex serves as a reservoir for recombinant DNase I in circulation [56]. The higher dosage used in our PK studies in rats should saturate the binding proteins and result in a larger proportion of DNase I in the unbound state.

We decided to evaluate rat plasma concentrations of the PASylated hyperactive DNase I at different sampling times using a bioassay for quantifying the intrinsic nuclease activity of the enzyme. In spite of its significant inhibition in serum, the catalytic activity of DNase I can be easily restored by sample dilution in assay buffer [69]. As result, even 96 h after i.v. injection, the bioactive enzyme was detectable much above background in rat plasma samples by this assay, which was in agreement with the prominent staining of the intact fusion protein on a Western blot (Figure 4b). Similar to the published PK study regarding Dornase alfa in rats [56], the concentration vs. time plot for the PASylated hyperactive DNase I was characterized by a bi-exponential decay, in accordance with a first-order elimination process. However, compared to native DNase I, the clearance of the PASylated enzyme took much longer: after the distribution phase, half of the drug was eliminated within 17 h (Table 3), which corresponds to a 6-fold increase in the half-life in rodents. This substantial 22-fold decrease in the systemic clearance most likely results from the restricted passage through the glomerular filtration barrier [67], due to the increase in apparent molecular size effected by PASylation (Table 2). It is tempting to assume that the PK profile in humans will be similarly improved. Based on empirical interspecies relationships [70], this may translate into an expected half-life of 2 days for this engineered version of DNase I in man.

Encouraged by the prolonged survival observed for lupus-prone mice treated with DNase I [71], a first-in-human clinical study with single bolus i.v. administration of human DNase I was conducted as early as 1999 in patients suffering from autoimmune systemic lupus erythematosus [34]. Although well tolerated, no clinical benefit was observed in this group of patients, which the authors claimed was most probably caused by the short plasma half-life and, consequently, a lack of sufficient circulating concentration of bioactive DNase I [34,69]. Meanwhile, recombinant DNase I was successfully explored in preclinical studies as thrombolytic agent to target elevated levels of extracellular DNA, likely released as NETs, after ischemic stroke [10,11,13,72,73]. This prompted translation into the clinic, where the efficacy of intravenous Dornase alfa (500 µg/kg) to ameliorate systemic inflammatory consequences after stroke is currently investigated in phase 2 trials (NCT04785066; NCT05203224; NCT05880524; https://clinicaltrials.gov, accessed on 4 May 2024). However, the present formulation of Dornase alfa was optimized for the daily treatment of lung diseases via inhalation therapy, which should limit its repurposing for intravenous administration. Hence, for pathological conditions where a sustained blood concentration of DNase I is needed to restore tissue balance, the therapeutic use of the PASylated hyperactive DNase I with its prolonged circulation and enhanced action should offer a versatile alternative.

## 5. Conclusions

Neutralization of DNA by in vivo DNase treatment has been shown to enhance the breakdown of clots. Herein, by engineering the physical size and active site of human DNase I, we were able to significantly enhance the bioavailability of this therapeutic enzyme in the body. Compared to the natural DNase I, the improved variant fused with the PAS-biopolymer shows a much higher hydrolytic activity towards DNA and has a considerably prolonged pharmacokinetic profile after a single intravenous dose in a preclinical model. Translated to humans, we expect that this candidate has the potential to replace the current clinical formulation of DNase I, which was approved for once-daily inhaled application and, thus, is not optimal for intravenous administration.

## Figures and Tables

**Table 1:** Productivity of the best-producing clones for PASylated hyperactive DNase I as assessed from 30 mL fed-batch cultures in shake flasks after 14 days. Product titers were quantified using the PicoGreen DNase I activity assay. Protein titers (mass of the PAS polypeptide included) were calculated by a standard curve with known concentration. Cell-specific productivity is defined as time-dependent increase in protein product concentration and normalized to the VCD from day 0 to day 14.

Rank	Clone Number	Product Titer [mg L^−1^]	Cell-Specific Productivity [pg d^−1^]
1	137	438	9.3
2	133	429	9.2
3	75	402	7.1
4	144	311	5.3
5	26	238	4.3
6	85	238	4.7
7	83	237	4.8
8	43	211	4.6

**Table 2:** Comparison of the molecular size between Dornase alfa and PASylated hyperactive DNase I analyzed by SEC and DLS. DLS data were derived from cumulants analysis and are given as mean peak size of three independent results ± corresponding standard deviation.

	Molecular Size	
	Dornase Alfa	PASylated DNase I
Amino acid residues	260	662
Protein mass [kDa], calculated	29.254	62.447
SEC apparent size [kDa] ^1^	38	581
DLS estimated MW [kDa]	39 ± 1.31	494 ± 10.6
DLS Z-average mean size [nm]	5.7 ± 0.11	16.9 ± 0.78

^1^ The discrepancy between calculated and apparent molecular size of unmodified DNase I can be explained by its post-translational N-glycosylation [3], which also contributes to the hydrodynamic volume, whereas the additional large increase in apparent size for the PASylated enzyme is due to the random coil effect.

**Table 3:** Primary and secondary PK parameters of PASylated hyperactive DNase I in rats following single i.v. administration of 5.33 mg per kg b.w.

Parameter ^1^	Unit	Value	SE ^2^	CV% ^3^
AUC_0→∞_	h µg mL^−1^	2616	47	1.78
$\mathrm{Initial}\;\mathrm{half}\text{-}\mathrm{life}\;t_{1/2}^{\alpha }$	h	5.9	2.3	39.50
$\mathrm{Terminal}\;\mathrm{half}\text{-}\mathrm{life}\;t_{1/2}^{\beta }$	h	17.2	0.7	4.02
C_max_	µg mL^−1^	130.0	4.2	3.21
V_SS_	mL kg^−1^	46.4	1.0	2.23
C_L_	mL h kg^−1^	2.04	0.04	1.78
MRT	h	22.8	0.31	1.36

^1^ AUC, area under the curve; C_max_, peak concentration in blood; V_ss_, steady state volume of distribution; C_L_, clearance; MRT, mean residence time. ^2^ SE, standard error. ^3^ CV%, coefficient of variation.

## Data Availability

The data presented in this study are available in the research article.

## Supplementary Materials

The following supporting information can be downloaded at: https://www.mdpi.com/article/10.3390/pharmaceutics16070967/s1.
