# Supplementary material for: Development of a Clonal and High-Yield Mammalian Cell Line for the Manufacturing of a Hyperactive Human DNase I with Extended Plasma Half-Life Using PASylation® Technology
Source: Pharmaceutics. 2024 Jul 22;16(7):967. doi: 10.3390/pharmaceutics16070967 (PMC11280007; doi:10.3390/pharmaceutics16070967)
Supplement: Supplementary file 1 [file pharmaceutics-16-00967-s001.zip › pharmaceutics-3059119-supplementary.pdf]

Article

Development of a clonal and high-yield mammalian cell line for the manufacturing of a hyperactive human DNase I with extended plasma half-life using PASylation® technology

Serge M. Stamm [1], Roland Wagner [1], Dietmar A. Lang [1], Arne Skerra [2,3]* and Michaela Gebauer [2]*

[1] Rentschler Biopharma SE, Erwin-Rentschler-Str. 21, 88471 Laupheim, Germany
[2] XL-protein GmbH, Lise-Meitner-Str. 30, 85354 Freising, Germany
[3] Lehrstuhl für Biologische Chemie, Technische Universität München, Emil-Erlenmeyer-Forum 5, 85354 Freising, Germany

* Correspondence: gebauer@xl-protein.com (M.G.); skerra@tum.de (A.S.); Tel.: +49 8161 714351 (A.S.)

Original Images for Gels and Blots

Original Gel image, Figure 1:

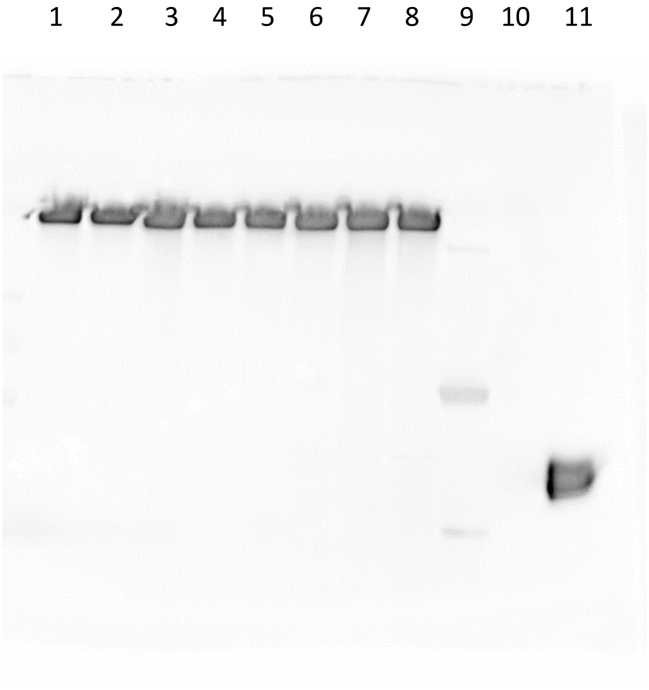

Figure S1. Western blot analysis of crude cell supernatants from stably transfected CHO TurboCell™ clones secreting recombinant PASylated hyperactive DNase I. Fed-batch cell culture supernatants after 14 days of cultivation (30 mL) separated on a reducing SDS-PAGE and blotted onto a PVDF membrane. DNase I was detected by a rabbit anti-human DNase I antibody, followed by an HRP-conjugated goat anti-rabbit antibody. Lanes 1-8: 1 µL of culture supernatant; lane 9: 1 µL trastuzumab culture supernatant (negative control); lane 10: 1 µL untransfected TurboCell™ (mock) culture supernatant; lane 11: 25 ng Dornase alfa. Note that PASylated proteins migrate in SDS-PAGE at an apparently higher molecular weight than expected from their true mass [31]. N-linked carbohydrate heterogeneity at Asn18 and Asn106 in Dornase alfa causes the protein to migrate as multiple bands with an average MW of 37 kDa [3] while the molecular weight predicted from the amino acid sequence is 29.3 kDa. Background signals observed for the control (lane 9) can be attributed to cross reactivity of the secondary antibody with heavy and light chains of trastuzumab.

<u>Original Gel image, Figure 3 (a):</u>

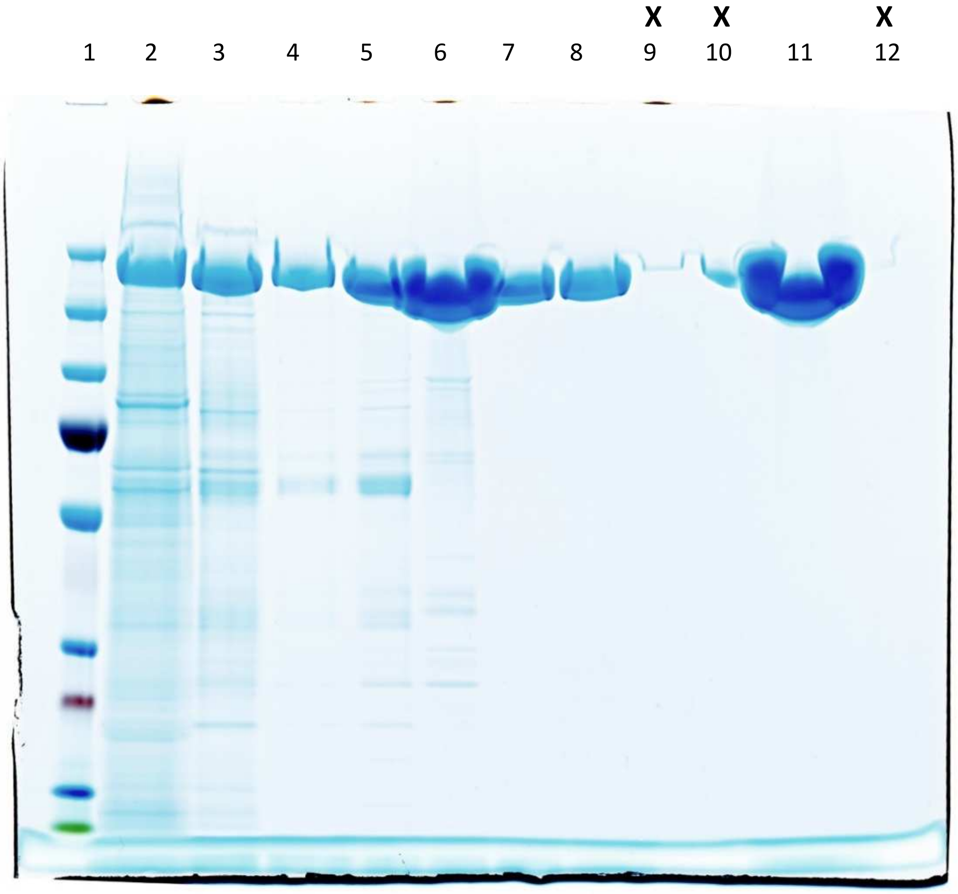

Figure S2 (a) SDS-PAGE analysis of the downstream purification process. Coomassie-stained reducing SDS-PAGE (4-12%). Lane 1: MW standard; lane 2: harvested cell culture supernatant from the bioreactor of clone 137; lane 3: AEX elution: lane 4: HIC elution; lane 5: CEX load after concentration and dialysis; lane 6: CEX flow-through (concentrated); lane 7: early flow-through from multimodal chromatography; lane 8: late flow-through; lane 9: — (no load); lane 10: — (no load); lane 11: concentrated protein solution of final protein preparation (63 μg); lane 12: — (no load).

X: lanes not included in the final figure

Original Blot image, Figure 4 (b):

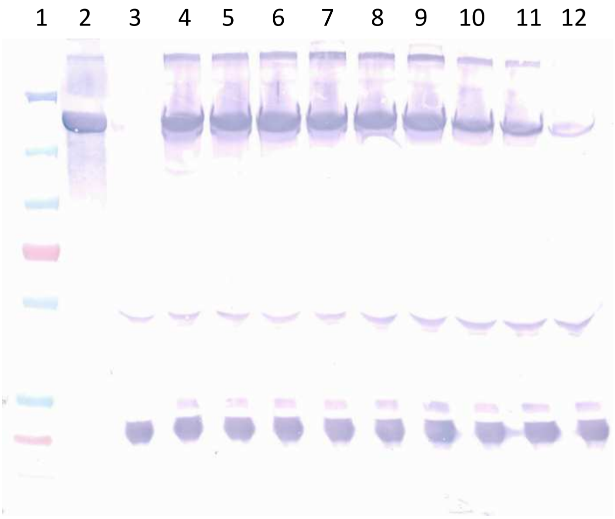

Figure S3 (b) Western blot analysis of rat plasma samples obtained at various sampling times after i.v. injection of PASylated DNase I in 4 (a). Equal volumes of rat plasma were subjected to western blotting after separation by reducing SDS-PAGE. PASylated DNase I was detected by a murine anti-PAS Mab followed by a goat anti-mouse IgG (H+L) AP-conjugate. Lane 1: pre-stained molecular size standard; lane 2: purified PASylated DNase I (control); lane 3: plasma sample of untreated rat (blank); lanes 4-12: rat plasma samples at 0.5 h, 1 h, 2 h, 4 h, 8 h, 12 h, 24 h, 48 h and 96 h, respectively. Each time point represents a mix of plasma collected from three animals.
